# 7q11.23 Microduplication Syndrome: Clinical and Neurobehavioral Profiling

**DOI:** 10.3390/brainsci10110839

**Published:** 2020-11-11

**Authors:** Maria Lisa Dentici, Paola Bergonzini, Francesco Scibelli, Cristina Caciolo, Paola De Rose, Francesca Cumbo, Viola Alesi, Rossella Capolino, Ginevra Zanni, Lorenzo Sinibaldi, Antonio Novelli, Marco Tartaglia, Maria Cristina Digilio, Bruno Dallapiccola, Stefano Vicari, Paolo Alfieri

**Affiliations:** 1 Medical Genetic Unit, Bambino Gesù Children’s Hospital, IRCCS, 00165 Rome, Italy; marialisa.dentici@opbg.net (M.L.D.); rossella.capolino@opbg.net (R.C.); lorenzo.sinibaldi@opbg.net (L.S.); mcristina.digilio@opbg.net (M.C.D.); 2 Genetics and Rare Diseases Research Division, Bambino Gesù Children’s Hospital, IRCCS, 00165 Rome, Italy; ginevra.zanni@opbg.net (G.Z.); marco.tartaglia@opbg.net (M.T.); 3 Child and Adolescent Neuropsychiatry Unit, Department of Neurological and Psychiatric Science, Bambino Gesù Children’s Hospital, IRCCS, 00165 Rome, Italy; paola.bergonzini@opbg.net (P.B.); francesco.scibelli@opbg.net (F.S.); cristina.caciolo@opbg.net (C.C.); paola.derose@opbg.net (P.D.R.); francesca.cumbo@opbg.net (F.C.); stefano.vicari@opbg.net (S.V.); 4 Laboratory of Medical Genetics, Bambino Gesù Children’s Hospital, IRCCS, 00165 Rome, Italy; viola.alesi@opbg.net (V.A.); antonio.novelli@opbg.net (A.N.); 5 Unit of Neuromuscular and Neurodegenerative Disorders, Department of Neuroscience, Bambino Gesù Children’s Hospital, IRCCS, 00165 Rome, Italy; 6 Scientific Directorate, Bambino Gesù Children’s Hospital, IRCCS, 00165 Rome, Italy; bruno.dallapiccola@opbg.net; 7 Department of Life Sciences and Public Health, Catholic University, 00168 Rome, Italy

**Keywords:** dup7q11.23, duplication, Williams–Beuren Syndrome, anxiety disorder, intellectual disability, congenital anomalies

## Abstract

7q11.23 Microduplication (dup7q11.23) syndrome is a rare autosomal dominant disorder due to a recurring 1.5 to 1.8 Mb duplication of the Williams–Beuren Syndrome critical region. Dup7q11.23 has been associated with several neuro-behavioral characteristics such as low cognitive and adaptive functioning, expressive language impairment, anxiety problems and autistic features. In the present study, we analyze the clinical features of ten individuals in which array-CGH detected dup7q11.23, spanning from 1.4 to 2.1 Mb. The clinical characteristics associated with dup7q11.23 are discussed with respect to its reciprocal deletion. Consistent with previous studies, we confirm that individuals with dup7q11.23 syndrome do not have a homogeneous clinical profile, although some recurring dysmorphic features were found, including macrocephaly, prominent forehead, elongated palpebral fissures, thin lip vermilion and microstomia. Minor congenital malformations include patent ductus arteriosus, cryptorchidism and pes planus. A common finding is hypotonia and joint laxity, resulting in mild motor delay. Neuropsychological and psychodiagnostic assessment confirm that mild cognitive impairment, expressive language deficits and anxiety are recurring neurobehavioral features. New insights into adaptive, psychopathological and neurodevelopmental profiles are discussed.

## 1. Introduction

Interstitial deletions of 7q11.23 cause Williams–Beuren Syndrome (WBS) (MIM 194050). Most deletions are de novo, while inherited deletions are very rare [1,2]. WBS is a distinct neurodevelopmental disorder easily recognized on the basis of a few key features, such as a characteristic facial gestalt, supravalvar aortic stenosis (SAS), prenatal growth deficiency, failure to thrive in infancy and intellectual disability (ID) [3,4]. Sleep disorders, including increased sleep latency and decreased sleep efficiency, occur in 65% of these patients [5]. Reduced grey matter volume in the intraparietal sulcus, occipito-parietal sulcus, brain stem and occipital lobe regions have been detected consistently by brain magnetic resonance imaging (MRI) [6]. Of note, seizures rarely occur [7].

More recently, the reciprocal microduplication of the same chromosomal region implicated in WBS has been identified in several patients who underwent array comparative genomic hybridization analysis (array-CGH) because of ID, language impairment, mild facial dimorphisms and, less frequently, congenital malformations [8,9,10]. Parental transmission of dup7q11.23 is quite common [11]. Unlike its reciprocal deletion, dup7q11.23 is not associated with distinct clinical features. However, some recurring dysmorphic features have been reported, including broad forehead (46%), straight brow line (43%), deep set eyes (33%), high/broad tip (56%), abnormal columella (78%), thin upper lip (64%), and short philtrum (59%) [10]. Macrocephaly occurs in 30–50% of affected individuals [9,10], while 17% of cases display short stature [12]. Dilation of the ascending aorta (DAA) and patent ductus arteriosus (PDA), occurring in approximately 45% and 15% of individuals, respectively, are the most common cardiovascular defects [12]. Seizures are found in 17% of cases and cryptorchidism in 15% of the male population [8,12,13].

To the best of our knowledge, very little information has been collected on the sleep pattern of children with dup7q11.23 syndrome. About 80% of reports have documented abnormal brain MRI, including ventriculomegaly, thin corpus callosum, increased extra-axial spaces, thin white matter, delayed myelination, posterior fossa cysts, and cerebellar vermis hypoplasia [10,12,13,14,15]. However, no recurring distinguishing feature has been reported [10].

Abnormalities in collagen due to an abnormal expression of the *ELN* gene is found both in WBS and dup7q11.23 syndrome [16,17].

A considerable number of studies have explored the neurobehavioral features of individuals with WBS [3,18,19,20,21,22,23,24]. Several of them have reported mild to severe ID with severely impaired visual spatial abilities, impaired receptive language skills and relatively spared expressive language skills [3,22,23,24,25]. Some investigations have reported that anxiety problems occur in 16.5% to 82.2% of WBS individuals [26,27]. Individual with WBS are well known for their enhanced social motivation (i.e., “hypersociable behaviors”) [3,24]. Nonetheless, some recent studies have highlighted socio-communicative problems in children with WBS, including deficits in shared attention, pragmatic use of language, the understanding of social relationships and emotion comprehension. [24,28]. Thus, enhanced social motivation does not implicate better social skills in individuals with WBS.

Relatively few studies have investigated the neurobehavioral profile of children with dup7q11.23 syndrome [8,9,29,30]. Studies on cognitive and developmental levels reported variable scores ranging from moderate ID/global developmental delay (DD) to average cognitive/developmental level [8,29,30]. The adaptive level of children with dup7q11.2 is usually below the normal range (mild to moderate impairment), and generally lower than their intelligence quotient [9,30,31,32].

Some studies have highlighted the presence of both receptive and/or expressive language deficits, as well as oral motor and vocal sound disorders [13,25,29]. Some of these studies suggest that expressive skills are more severely affected than receptive ones [25,29]. Psychopathological features have also been investigated in past research, reporting that most children with dup7q11.23 syndrome meet the criteria for diagnosis of specific phobias and/or social anxiety [9,29]. More recently, autism spectrum disorder (ASD) [9,30] has been considered a relatively common feature in children with dup7q11.23 syndrome (33%) [9]. However, the use of gold standard diagnostic tools combined with clinical judgments disclosed a lower presence of ASD in this population (around 19%) [30]. The discrepancy between diagnostic tools (parental report vs. clinician report) could be partially explained by the severe social anxiety described in several studies [9,29] which could mime autism-like behaviors [30].

Past studies have contributed to characterizing the phenotype of dup7q11.23 individuals, but only a few of them have comprehensively assessed the neurobehavioral features in representative patients’ samples [9]. Some neuropsychological features, such as visual–motor skills, have not yet been investigated. Therefore, additional studies are warranted in order to extend the available data on neurobehavioral features of dup7q11.23 individuals.

In this study, ten patients with dup7q11.23 identified by array-CGH are described. We provide data on clinical features, cognitive level, adaptive profile, language skills, behavioral problems and psychopathological features. We discuss the present results with published data of patients with duplication and deletion (WBS) 7q11.23 syndromes, in order to outline distinguishing and shared clinical features.

## 2. Materials and Methods

### 2.1. Participants

The cohort included ten children with typical dup7q11.23. There were three females and seven males. The mean (M) age was 97.2 months (Standard Deviation (SD): 30.07). All participants were recruited at the Child and Adolescent Psychiatry Unit and Genetics and Rare Diseases Research Division of the Bambino Gesù Children’s Hospital, Rome.

### 2.2. Cytogenetics and molecular cytogenetics

DNA was extracted from whole peripheral blood by means of Qiagen blood and tissue kit (https://www.qiagen.com), according to the manufacturer’s instructions. CMA (chromosomal microarray analysis) was performed using the Agilent Oligo arrays 8 × 60K (patients 1, 2, 3, 6, 8 and 9), Agilent Oligo arrays 4 × 180K (patients 4, 5, 10) and Illumina CytoSNP 850K (patient 7), using standard procedures. Images were obtained using an Agilent DNA Microarray Scanner and Agilent Scan control Software (v A.8.4.1), and analyses were performed by Agilent CytoGenomics (v 4.0.3.12) or Illumina BlueFuse Multi (v 4.4), depending on the platform. Validation of the genomic rearrangement and segregation analysis was performed by FISH (fluorescence in situ hybridization) on metaphases obtained by lymphocyte cultures from the patients and their parents (when available). Vysis Williams Region Probe–LSI ELN was used for this purpose (https://www.molecular.abbott/int/it/vysis-fish-chromosome-search).

### 2.3. Neurobehavioral Assessment

Cognitive Level: Cognitive level was assessed by means of appropriate developmental tools—Wechsler Intelligence Scale for Children—Fourth Edition (WISC-IV) [33] and Leiter International Performance Scale-Third Edition (Leiter-3) [34]. WISC-IV is a tool utilized to evaluate cognitive abilities in patients older than 6 years. The test provides four indices: verbal comprehension index (VCI), perceptual reasoning index (PRI), working memory index (WMI), speed processing index (PRI) plus a full scale intelligence quotient (FSIQ). When participants showed severe communicational impairment, Leiter-3 was used to assess the non-verbal cognitive profile. Leiter-3 provides for a non-verbal intelligence quotient (NVIQ).

Adaptive level: The adaptive level was assessed by using Vineland Adaptive Behavior Scales—Second Edition (VABS II) [35], a semi-structured interview with the caregiver. VABS II provides four domains (communication, daily living skills, socialization and motor skills). Adaptive behavior composite (ABC), a global index of adaptive behavior, corresponds to the sum of four domains. Only in one case was Adaptive Behavior Assessment System—Second Edition (ABAS II) [36], a parent-report tool for adaptive behavior measurement, administered. ABAS II provides a composite adaptive behavior score, and four domains: conceptual, social and practical. Although we used different tools for the adaptive functioning, it was possible to combine the scores referring to similar domains given the high correlation between domains of tools [36].

Lexical Production Skills: Lexical production was evaluated by means of the following developmentally appropriate tools:

1. Expression subtest of the PhonoVocabulary Test (TFL, Test Fono-Lessicale) [37]. The expression subtest contains 45 tables. The examiner indicates a picture on the table and asks the participant to label the targeted picture;
2. The Boston Naming Test [38]. In this test, the participant is asked to label the name of each shown picture. Participants have about 20 s to answer each question;
3. Expression subtest of “Batteria per la Valutazione del Linguaggio 4-12” (BVL 4–12) [39]. In the expression subtest, participants are required to label 67 images (51 nouns and 16 verbs);
4. Expression subtest of “Parole in Gioco” (PinG) [40]. In this subtest, the child must name the pictures shown.

Lexical Comprehension Skills: Lexical comprehension was evaluated by means of the following developmental appropriate tools:

1. Receptive subtest of PhonoVocabulary Test (TFL, Test Fono-Lessicale) [37]. The receptive subtest contains 45 tables with four pictures. Participants must choose the picture labeled by examiner;
2. Peabody Picture Vocabulary Test–PPVT [41]. In this test the examiner says a word describing one of four drawings and asks the participant to point to the labeled drawing. Raw total score is converted into Lexical Quotient (LQ);
3. Receptive subtest of BVL 4-12 [39]. In this test the examiner says a word describing one of four images and asks the participant to point to the labeled images. This subtest contains 42 words (31 nouns, 10 verbs, and 1 adjective);
4. Receptive Subtest of Parole in Gioco (PinG) [40]. In this subtest three pictures are shown and the subject must point to the picture corresponding to the word labeled by the examiner.

Grammar Comprehension Skills: Grammar comprehension skills were evaluated by using the following developmental appropriate tools:

1. Grammar Evaluation Test (PVCL, Prove di Valutazione della Comprensione Linguistica) [42]. In this test, the examiner pronounces a sentence describing one of four pictures. Then, the examiner asks participants to choose the correct picture;
2. Grammar Evaluation Subtest of BVL 4-12 [39]. In syntactic comprehension subtest, children are asked to recognize each of the 40 examiner’s sentences within one of four pictures.

Visuomotor integration skills: Visuomotor integration skills were investigated by means of the Beery–Buktenica Developmental Test of Visual–Motor Integration (VMI) [43]. In this assessment, the child is required to copy some drawings. The drawings are shown in an increasingly difficulty order. Use of perception (VP) and motor coordination (MC) subtests allow us to assess one skill set, excluding the other.

Behavioral/Psychopathological Problems (Parent Report): The parent-report version of the Child Behavior Checklist (CBCL) [44] was used to measure problem behavior. Both Child Behavior Checklist 1½–5 (CBCL 1½–5) and Child Behavior Checklist 6–18 (CBCL 6–18) were used. Item scoring provides the following subscales: Anxious/ Depressed, Withdrawn/ Depressed, Somatic Complaints, Social Problems, Thought Problems, Attention Problems, Rule-Breaking Behavior, Aggressive Behavior. Furthermore, three composite scales can be computed (Externalizing, Internalizing and a Total Problem). Conners’ Parent Rating Scales-Long Version, Revised (CPRS-R-L) [45], is a parent-report questionnaire that is administered to assess attention deficits and hyperactivity disorders (ADHD) symptoms as well as other symptoms of behavioral and emotional disorders commonly associated based on DSM-IV-TR [46] criteria.

Behavioral/Psychopathological Problems (Clinician Report): Clinical history and actual psychiatric symptomatology were investigated through unstructured interviews performed by a Child Neuropsychiatrist and Licensed Clinical Psychologist. When applicable, the semi-structured interview Schedule for Affective Disorders and Schizophrenia for School-Age Children-Present and Lifetime Version (K-SADS-PL) [47] was used to evaluate psychiatric comorbidities in our clinical group. Furthermore, the Children’s Global Assessment Scale (C-GAS) was utilized to assess the psychosocial function of the participants [48]. C-GAS scores (ranging from 1 to 100) is a measurement independent by specific diagnosis. Higher scores indicates better functioning. A best-estimate consensus diagnosis was established for participants by a multidisciplinary team (at least one child psychiatrist and one clinical psychologist) based on psychiatric evaluation, psychopathological/behavioral questionnaires and, when administrable, semi-structured interview [47]. Diagnoses were based on Diagnostic and Statistical Manual of mental disorders–Fourth Edition –Text revised (DSM-IV-TR) [46]. 

Autism symptoms (Parent report): Autism Symptoms were assessed by means of Social Responsiveness Scale (SRS) [49] a widely used parent report questionnaire that evaluates a child’s social awareness, cognition, communication, motivation, and mannerisms. A total score is also available. Higher scores indicate greater social communication difficulties. 

Autism symptoms (Clinician report): a clinician report assessment of autism symptoms was performed by means of Childhood Autism Rating Scale 2 (CARS-2) [50]. CARS-2 has 15 items scoring from 1 (normal behavior) to 4 (severely abnormal behavior). Clinician reports were based on the observation of the child and a parental questionnaire to assign scores. Higher total scores indicate more severe symptoms of autism.

### 2.4. Procedure

Patients were selected among children referred to our services between 2015 and 2020, because of psychomotor delay, and/or severe language impairment, and/or ASD and/or, more rarely, non-specific facial dysmorphisms. Only participants showing typical dup7q11.23 confirmed by FISH were included in this study. Nine of the ten selected participants were evaluated through neurobehavioral assessment tools. The study was approved by the ethics committee of the Bambino Gesù Children Hospital, Rome (approval number 590). All participating patients/families provided signed informed consent. 

## 3. Results

Results are summarized in Table 1, Table 2, Table 3 and Table 4, and additional information is available in Supplementary Materials.

### 3.1. Cytogenetics and Molecular Cytogenetics Analyses

Ten patients with dup7q11.23 were grouped out, their features were compared to one another’s and to the clinical features commonly reported in this syndrome (OMIM # 609757, https://omim.org/). 

**Figure 1:**
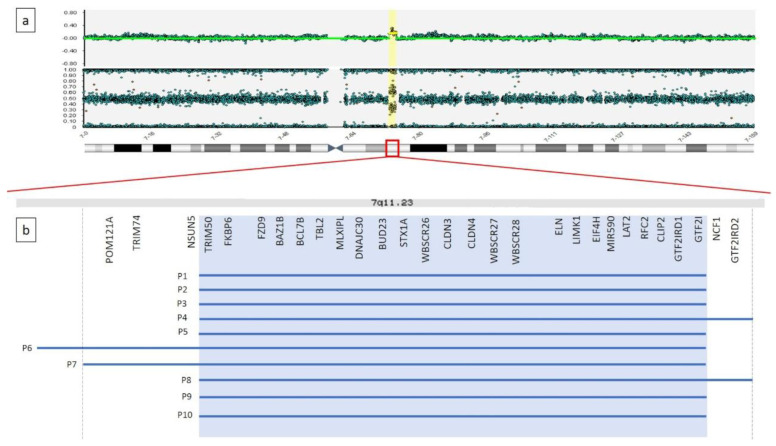
(**a**) Graphical representation of SNP-array result for patient 7. A microduplication is detected on the long arm of a chromosome 7, at 7q11.23, 1.9 Mb in size. (**b**) Enlargement of 7q11.23 genomic region. Gray background shadow highlights the OMIM genes normally included within the recurrent breakpoints reported for the microduplication syndrome. Blue lines represent the microduplications detected in our 10 patients.

The duplications spanned 1.4 Mb to 2.1 Mb, and all involved the core region reported as critical for the duplication syndrome. Of note, 4 out them presented with atypical breakpoints, spanning proximally or distally the recurrent ones. In particular, the duplication detected in P4 and P8 extended towards the telomere, involving two further OMIM genes *NCF1* and *GTF2IRD2*, while the ones detected in P6 and P7, expanding towards the centromere, involved the extra OMIM genes *NSUN5*, *TRIM74* and *POM121A*. Details about breakpoints and gene content are summarized in Table 1 and Figure 1.

### 3.2. Clinical Features

**Figure 2:**
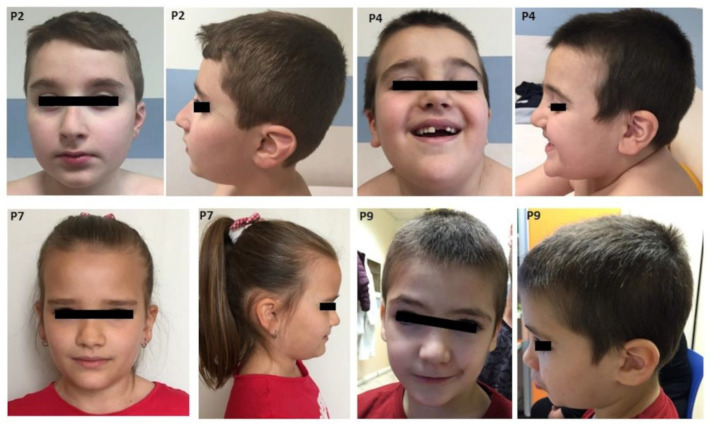
Photographs of patients with a 7q11.23 microduplication (P2, P4, P7, P9). Note macrocephaly, prominent forehead in some patients, elongated palpebral fissures, deep set eyes, palpebral ptosis, high prominent nasal bridge with bulbous nasal tip, short philtrum and thin vermillion of the lips.

Table 1 summarizes the cytogenetic, clinical and developmental findings in the present cohort of dup7q11.23 individuals. Figure 2 illustrates the facial features of patients 2, 4, 7 and 9.

Growth and nutrition: Growth parameters were within normal range in the majority of the individuals and in none of them height was below the 3rd centile. 

Facial features: Recurring facial features included macrocephaly (3/8), prominent forehead (6/8), elongated palpebral fissures (7/10). Prominent nose was a common finding, either with a high nasal bridge or a bulbous nasal tip (7/8). Six individuals had thin lip vermilion and three microstomia. 

Hearing loss: Hearing loss was detected only in one individual presenting a mild conductive defect due to tubal stenosis. 

Congenital heart defects: Congenital heart disease was present in 5/10 patients, including PDA (3/10), pulmonary valve dysplasia (1/10), mild DAA (1/10), mild tricuspid insufficiency (1/10), left ventricular hypertrabeculation and mild aortic insufficiency (1/10). 

Musculoskeletal and motor milestone: Both hypotonia and joint laxity were quite common (4/10), resulting in mildly delayed achievement of motor milestones. Four patients presented pes planus (4/10).

Genitourinary system: Features also included cryptorchidism (2/7).

Minor clinical features: Other minor clinical features are listed in Table 1, including periodic migraine (2/10), and single cases with refractive errors (hypermetropia and astigmatism), hepatic steatosis, asthmatic bronchitis and bronchospasm. 

Sleep problems: Only one individual reported an irregular sleep pattern resolved after the age of 4. 

Brain malformations: A brain MRI was performed on four individuals who did not have any distinct malformation. Thin corpus callosum was recorded in two of them.

Cognitive level: Neurobehavioral results are illustrated in Table 2, Table 3 and Table 4. No participant had a normal cognitive level, the results ranging from borderline to mild delay; only one child displayed moderate cognitive impairment. 

Adaptive functioning: All participants had an adaptive behavior level below the normal range. The adaptive level tanged from mild to moderate delay, usually being below the cognitive level of the children. An analysis of adaptive relative strengths and weakness showed that the Socialization and Daily Living Skills domains were relatively higher compared to the Communication domain. Language skills: Results on language assessment disclosed global impairment of lexical production and comprehension skills, and grammar comprehension skills in the majority of the children, only one of them having spared lexical production and comprehension skills.

Visuo-motor integration skills: Visuo-motor integration skills were average in 3 of 9 children, while 6 had scores below average. The total sample ranged from borderline to moderate level (M = 75.22; SD = 18.59).

Behavioral problems: Analysis of questionnaires revealed several emotional, behavioral, and attention problems. CBCL (available in 8 participants) [44] showed borderline/clinical results in “Withdrawn/ Depressed” in 50% of children in the study, and “Anxious/ Depressed” and “Social Problems” scales in 37.5% of them. “Internalizing problems” reached the borderline/clinical score in 50% of children. CPRS-R-L (available in 8 participants) [45] revealed that “Cognitive problems/inattention”, “Anxious–shy”, “Perfectionism”, “Social Problems”, “DSM–IV inattentive”, and “DSM–IV total” were in borderline/clinical range in half of the participants.

Psychiatric diagnosis and functioning: The most recurring psychopathological diagnoses assigned by best estimate consensus diagnosis were “Anxiety Disorders” (DSM IV-TR) [46] in 78% of participants. Analysis of C-GAS (available in 5 children) [48] showed a “moderate degree of impairment” in 3 participants, while two subjects had “variable functioning” and “major impairment”.

Autism symptoms: Evaluation of autism symptoms assessed by parent report tool disclosed in 71% of participants mild to severe impairment in at least one scale of SRS [49]. However, only one child met the cut-off for ASD in the clinician report tool CARS-2 [50] even if he does not reach full criteria for ASD by following best estimate consensus diagnosis.

## 4. Discussion

The present study provides detailed clinical description of ten subjects with dup7q11.23. This rearrangement results in a mild non-characteristic phenotype, thus preventing the possibility to reach the diagnosis based on clinical criteria only [13,51]. However, a few facial features are recurring, including macrocephaly, prominent forehead, deep set eyes, elongated palpebral fissures, short philtrum and thin lips (see Table 1). In general, our results support previous observations [9,10]. The duplications were extended from 1.4 Mb to 2.1 Mb and they all involved the core region reported as critical for the duplication syndrome. Four patients presented atypical breakpoints, spanning proximally or distally the recurrent region. In particular, the duplication detected in P4 and P8 extended towards the telomere, involving *NCF1* and *GTF2IRD2* genes, while the ones detected in P6 and P7, expanding towards the centromere, involved the *NSUN5*, *TRIM74* and *POM121A genes* (Table 1 and Figure 1). No other CNV of significant relevance was found. Physical examination of the patients with a larger duplication were compared to the subjects with the duplication spanning the core critical region (1.4 Mb) and no gross phenotypic differences were highlighted (Table 1). Of note, P7 presented only mild facial dimorphism (Figure 1) and no congenital malformation beside PDA, which was the most recurrent coronary heart disease (CHD) reported in P5 and P9 that present the typical 1.4 Mb recurrent microduplication. Some facial features can be regarded as countertypes of periorbital fullness, short nose, long smooth philtrum and thick vermilion of the upper and lower lips found in children with WBS. In WBS, the most common vascular disease is SAS, followed by peripheral pulmonic stenosis that is common in infancy but usually improves over time [4]. The most common cardiovascular disease of dup7q11.23 is DAA, occurring in 46% of the patients [12]. We found DAA in one subject only, while PDA was the most common cardiovascular disease in the present series of patients. The frequency of PDA overlaps previous studies [12]. Hypotonia and joint laxity were common in our patients. This is not surprising considering the abnormal gene dose of ELN in deletion and dup7q11.23 syndromes [16,17].

As far as we know, this is the first study of sleep problems in dup7q11.23 children, which has excluded the presence of major problems, differently from what is generally observed in individuals with WBS [5]. In addition, none of our patients manifested epileptic seizures, a rare complication also in WBS [7].

WBS is characterized by prenatal growth deficiency, followed by a poor linear growth in the first years of life, a normal rate of linear growth in 75% of cases during childhood, and a mean adult height below the third centile. Our dup7q11.23 patients displayed a normal linear growth, differently from published data pointing to short stature in about 17% of cases [12].

We also confirm a rare association with hearing loss, which is reported in 5% of dup7q11.23 individuals [12], a figure much lower compared to WBS in which mild to moderate progressive sensorineural hearing loss occurs in about two thirds of pediatric cases [52]. Our data on cryptorchidism support previous findings [12,13], suggesting a slightly higher association in subjects with duplication compared with those with deletion [53]. Differently from WBS children [54], present results corroborate a grossly normal brain structure in dup7q11.23 subjects, apart for the occasional occurrence of thin corpus callosum [12].

In agreement with previous studies [13], and differently from WBS [1,2], we found parental transmission of the genomic imbalance in 3/8 families. Retrospective evaluation of heterozygous parents disclosed variable developmental delays with learning difficulties and language impairment in infancy.

Neurobehavioral phenotype 

All except one of our children displayed mild cognitive impairment. Available data suggests that cognitive impairment in children with dup7q11.23 syndrome is less severe than in WBS. However, comparative studies on larger patients’ cohorts are needed to support this conclusion.

All our subjects had adaptive profiles below their cognitive level. Adaptive behavior is defined as “the collection of conceptual, social, and practical skills that are learned and performed by people in their everyday lives” [55]. The analysis of adaptive relative strengths and weakness provided some interesting insights. In particular, the Communication domain appears to be a severely impaired area differently from previous studies who which showed that “Communication and Socialization” domains were relative strengths [9]. As far as we know, this is the first study reporting separate data on adaptive Communication and Socialization domains, previous studies used combined scales with a common domain for both skills [9]. Our results support that Socialization is a relative strength, even if below the normal range [9]. Weakness in adaptive Communication domain of children with dup7q11.23 enrolled in the present study differs from results obtained in children with WBS, where Communication is usually a relative strength above other domains [23,56,57,58].

The present data on adaptive communication are not unexpected, given that language impairment is invariably reported in published studies [12,29]. We found that lexical production, lexical comprehension, and grammar comprehension were impaired in the majority of our children. Since we used different assessment tools, we cannot provide any direct comparison of lexical production and lexical comprehension results.

Data on visual–motor integration have shown visual–motor skills at borderline level. As far as we know, this is the first study providing this assessment in dup7q11.23 children, differently from WBS, in which visual motor major impairments have been assessed and documented [59,60].

We found that about half of our children displayed anxiety, social and attentional problems based on parent-report tools. However, best estimate consensus diagnoses have shown that Anxiety Disorders are very common, while Disruptive Behavior Disorders Not Otherwise Specified are less common. In agreement with previous studies, we found that Anxiety Disorders affected more than 75% of dup7q11.23 patients [22]. Furthermore, the prevalence of Anxiety Disorders overlaps with the results obtained in children with WBS [26,27].

Surprisingly, despite several participants showing socially inhibited behaviors, none of our children met the full criteria for Social Phobia diagnosis. This is in contrast with previous results indicating about 50% of dup7q11.23 children having Social Anxiety [22]. In most cases, the qualitative analysis of psychiatric evaluations (Table 4) disclosed a combination of different symptoms that do not meet the criteria for specific disorders—anxiety/phobias, aggressive behaviors, separation anxiety/social inhibition, repetitive behaviors/movements, and social disinhibition. In agreement with previous studies [61], we suggest that some of these symptoms could be related in part to dysregulation of the oxytocin hormone. Past research [61] has identified associations between enhanced social motivation in WBS and elevated oxytocin, suggesting that the typical profile of this syndrome could be related to this neurohormone. Some authors [62] have highlighted that levels of oxytocin are positively associated with social approach and talking with strangers in an experimental environment, while they are negatively associated with social adaptive functioning. Furthermore, these authors found higher oxytocin levels in subjects with WBS when compared to control groups (approximately three times higher). On the contrary, in non-clinical populations, decreased oxytocin is linked to aggressive behaviors [63,64,65] and higher separation anxiety [66]. Thus, authors conclude that oxytocin could play a role in phenotypical expression of deletion vs. duplication syndrome. However, further experimental studies are needed to explore these hypotheses.

We found that 11% of our patients met the cut-off for ASD on the clinicians’ report tool, while a more significant proportion of children showed autistic-like behaviors, such as poor eye contact, repetitive behaviors and motor stereotypies, when evaluated by the self–parent tool. However, none of our children met the full diagnostic criteria for ASD following best estimate consensus diagnoses. Our data differ from previous figures on the prevalence of ASD in dup7q11.23 syndrome (19%) [67] and WBS (30/35%) [67,68]. While the use of different tools (CARS-2 [50] vs. Autism Diagnostic Observation Schedule—Second Edition [69]) could explain part of the difference in the prevalence of children who met instrument cut-offs [70], the present findings suggest a possible overestimation of ASD in dup7q11.23 individuals. The combination of symptoms like anxiety/phobias, separation anxiety/social inhibition, and repetitive behaviors/movements (Table 4) could have been inferred as ASD.

The present parental reports have highlighted that 44% of the children displayed socially disinhibited behaviors, such as hugging unfamiliar people (occasionally in alternation with social inhibited behavior) or showing sexual parts. So far, these behaviors have not been investigated in depth by using specific assessment tools. Future investigations should consider these tools for assessing social motivation.

The heterogeneous psychopathological and neurodevelopmental profiles emerging from the present study cast some doubt on the phenotype of dup7q11.23 syndrome as “opposite” to WBS. One could speculate that the psychopathological and neurodevelopmental heterogeneity could be associated in part with “social dysregulation” features. In fact, our children do not appear to be completely avoidant or disengaged from social relationships. These observations appear to be supported by the relative strength in socialization upon adaptive behavior evaluation. We suggest that children with dup7q11.23 might have difficulty in emotional regulation during social interaction, bringing them alternatively towards aggressive behaviors, social inhibition/separation anxiety or social disinhibited behaviors. However, further experimental studies on social motivation, social orientation and social engagement in these children are warranted to support this hypothesis.

## 5. Conclusions

In conclusion, the present study extends the available data on genotype–phenotype correlations in individuals with dup7q11.23, and provides new insight into the diagnosis, management and treatment of affected individuals. We confirm that facial dysmorphisms are not characteristic, although some recurring features, including macrocephaly, prominent forehead, elongated palpebral fissures, prominent nose, thin lip vermilion and microstomia, could provide some clues to diagnosis. We found that the adaptive and cognitive impairment of dup7q11.23 children is milder compared to WBS. The analysis of neurobehavioral profile has shown that the communication adaptive level is the most impaired domain compared to other adaptive domains. Differently from WBS, we found that dup7q11.23 individuals display only minor impairment in visual–motor integration, and confirm that language impairment is a major problem in most of the children studied so far. In agreement with previous studies, we confirm that the more common psychopathological issues include a wide spectrum of anxiety disorders, not necessarily social anxiety. The presence of isolated social disinhibited behaviors reported in some of our patients warrants further investigation in future studies. Finally, our data suggest that ASD could affect only a small proportion of dup7q11.23 individuals.

This study has some limitations, given the relatively small sample of evaluated subjects. In addition, participants were tested with different tools, based on their developmental level and compliance. Finally, carrier parents have not been assessed directly, since their retrospective evaluation was based on verbal reports. Future studies on larger number of patients are required to support our hypothesis on cognitive and adaptive profiles in dup7q11.23 children. Furthermore, cross-syndrome studies are warranted in order to compare the neurobehavioral patterns of children with 7q11.23 duplication and deletion syndromes.

## Figures and Tables

**Table 1:** Clinical features of our cohort of 10 patients affected by 7q11.23 microduplication syndrome.

Patient N	P1	P2	P3	P4	P5	P6	P7	P8	P9	P10	Total
**Gender**	M	M	F	M	F	M	F	M	M	M	7M; 3F
**Current Age (Years)**	7.7	9.10	5.7	5.6	4.9	12.3	9.2	7.5	7.8	11.3	M: 8; SD: 2.6
**Age at diagnosis (Years)**	3	6	3	3	1.6	8.6	7.6	3.6	4	11	M: 6.4; SD: 3.1
**CGH array** **(start and end point)**	7q11.23(72,726,578–74,119,570)x3	7q11.23(72,726,578–74,139,390)x3	7q11.23(72,726,578–74,139,390)x3	7q11.23(72,726,578–74,339,044)x3	7q11.23(72,726,578–74,139,390)x3	7q11.22q11.23(72,044,007–74,139,390)x3	7q11.23(72,283,565–74,134,911)x3	7q11.23(72,726,578–74,339,044)x3	7q11.23(72,726,578–74,119,570)x3	7q11.23(72,726,578–74,139,390)x3	
**Length of duplicated region**	1.4 Mb	1.4 Mb	1.4 Mb	1.6 Mb	1.4 Mb	2.1 Mb	1.9 Mb	1.6 Mb	1.4 Mb	1.4 Mb	1.4–2.1 Mb
**Inheritance**	*de novo*	maternal	NA	paternal	*de novo*	*de novo*	*de novo*	NA	paternal	*de novo*	5/8 *de novo*; 3/8 inherited
**Growth at last evaluation (centile)**	weight <3rd; height 3rd–10th; OFC 75th	weight >97th; height 90th–97th; OFC 50–75th	weight 10th; height 10th; OFC 25th	weight: 10th; height 97th; OFC >97th	Weight 50th–75th; height 25th–50th; OFC 75th–97th	Weight 50th– 75th; height 10th–25th	weight 50th-75th; height 25th–50th, OFC 50th	Weight:75th–97th; Height 25th–50th OFC 75th	weight: 75th; height 97th; OFC 75th	weight: 97th; height 50–75th	
**Facial**											
**Macrocephaly**	relative	no	no	yes	relative	NA	no	no	no	NA	3/8
**Brachycephaly**	no	yes	no	no	no	NA	no	yes	no	NA	2/8
**Prominent forehead**	yes	yes	no	yes	no	NA	yes	yes	yes	NA	6/8
**Elongated palpebral fissures**	yes	no deep set eyes	no	yes palpebral ptosis	no downslanting palpebral fissures	Yes	yes	yes deep set eyes	yes	yes palpebral ptosis	7/10 (2/10 ptosis; 2/10 deep set eyes)
**Nose**	high nasal bridge	high nasal bridge	high nasal bridge with bulbous nose	high broad nasal bridge	normal	NA	bulbous nose	prominent, high nasal bridge	bulbous nose	NA	5/8 high nasal bridge 3/8 bulbous nose
**Dysmorphic ears**	additional crus of the antihelix, abnormally folded helix	horizontal crus of helix	asymmetric low set and posteriorly rotated	low-set posteriorly rotated ears with thickened earlobes	thickened helix	NA	no	no	thickened helix large ears	NA	6/8
**Short philtrum**	no	yes	no	yes	no	prominent	yes	yes	yes	NA	6/9
**Microstomia**	yes	no	no	yes	yes	NA	no	NA	no	NA	3/7
**Thin lips**	yes	yes	yes	yes	yes, everted upper lip	No	no	no	yes	NA	6/9
**Other**	NA	telecanthus, medial flaring of the eyebrow, exophoria	horizontal eyebrow, synophrys, short neck, pectus excavatum	prognathism, long chin with horizontal crease	micrognathia	NA	horizontal eyebrow; short neck, widely spaced teeth	overhanging columella	synophrys, hypertelorism, divergent strabismus thickened nostrils	large incisors, short neck, low anterior and posterior hairline	
**Hearing loss**	no	no	no	no	no	mild conductive (tubal stenosis)	no recurrent otitis media	no	no	no	1/10
**Cardiac malformation**	no	mild ascending aortic dilatation	no	no	PDA, pulmonary valve dysplasia	No	PDA, mild tricuspid insufficiency	no	PDA(spontaneously resolved)	left ventricular hypertrabeculation, aortic insufficiency	3/10 PDA; 1/10 ascending aortic dilatation
**Cryptorchidism**	no	yes bilateral	NA	yes bilateral	NA	no	NA	no	no	no	2/7
**Hypotonia**	yes	no	yes	no	no	no	yes	no	no	yes	4/10
**Joint laxity**	yes	no	yes	no	no	no	yes	no	no	yes	4/10
**Pes planus-valgus**	yes	yes	yes	no	no	yes	no	no	no	no	4/10
**Neurological**											
**Brain MRI findings**	NA	Cerebral ultrasound: enlargement of periencephalic cerebral spaces	atrophy of EC with enlargement of the 3rd and lateral ventricles, thin corpus callosum	NA	NA	NA	normal	NA	normal	thin corpus callosum, arachnoid cyst	2/5 thin corpus callosum 3/5 aspecific abnormalities
**Epilepsy** **EEG**	normal	normal	abnormal	NA	NA	NA	normal	normal	normal	normal	1/7 abnormal
**Sleep pattern**	regular	regular	regular	NA	NA	irregular until 4 years	regular	regular	NA	NA	1/6 irregular
**Other**		periodic migraine episodes. hepatic steatosis		asthmatic bronchitis		Adenoidectomy; bronchospasm; pharmacological therapy (risperdone 0.25 mg; sertraline 0.5 mL).		hypermetropia astigmatism	pharmacological therapy risperdone 0.25 mg (2 times per day)	periodic migraine episodes, pelvis dilatation	2/10 migraine 1/10 hepatic steatosis

Legend: P: Patient; M: Male; F: Female; M: mean; SD: Standard Deviation; Mb: megabases; NA: not available; OFC: occipital frontal circumference; PDA: patent ductus arteriosus; MRI: Magnetic Resonance Imaging; EEG: electroencephalogram; EC: entorhinal cortex; mg: milligrams; ml: milliliters.

**Table 2:** Cognitive, adaptive, language and visuo-motor assessment.

Test	P1	P2	P3	P4	P6	P7	P8	P9	P10	Total
**Age in years**	7.7	9.10	5.7	5.6	12.3	9.2	7.5	7.8	11.3	M: 8.5; SD: 2.4
**Cognitive level**	85 ^a^	47 ^b^	82 ^a^	81 ^a^	75 ^b^	81 ^a^	65 ^b^	62 ^a^	68 ^a^	M: 71.77; SD: 12.39
**Adaptive Composite Score**	71 ^c^	51	57 ^c^	54 ^c^	62 ^c^	85 ^c^	45 ^c^	38 ^c^	40 ^d^	M: 55.88; SD: 15.13
**Communication Domain**	55 ^c^	56	45 ^c^	51 ^c^	64 ^c^	71 ^c^	45 ^c^	40 ^c^	49 ^d^	M: 52.88; SD: 9.84
**Daily Living Skills Domain**	82 ^c^	49	73 ^c^	58 ^c^	75 ^c^	107 ^c^	44 ^c^	42 ^c^	40 ^d^	M: 63.33; SD: 22.61
**Socialization Domain**	87 ^c^	65	81 ^c^	71 ^c^	59 ^c^	82 ^c^	67 ^c^	54 ^c^	53 ^d^	M: 68.78; SD: 12.46
**Motor Skills Domain**	-	-	54 ^c^	65 ^c^	-	-	-	-	-	M: 59.5; SD: 7.78
**Lexical Production**	severely impaired	severely impaired	severely impaired	severely impaired	spared	slightly impaired	slightly impaired	severely impaired	severely impaired	6/9 severely impaired 2/9 slightly impaired 1/9 spared
**Lexical Comprehension**	severely impaired	slightly impaired	severely impaired	severely impaired	spared	severely impaired	severely impaired	severely impaired	severely impaired	7/9 severely impaired 1/9 slightly impaired 1/9 spared
**Grammar** **Comprehension**	severely impaired	slightly impaired	severely impaired	severely impaired	severely impaired	severely impaired	severely impaired	severely impaired	severely impaired	8/9 severely impaired 1/9 slightly impaired 0/9 spared
**Visuo-motor skills**	73	74	57	111	58	92	89	63	60	M: 75.22; SD: 18.59

All scores are expressed in standard scores (Mean = 100; standard deviation: 15). Legend—P: patient; a: Leiter 3; b: WISC IV; c: VABS II; d: ABAS II; M: Mean; SD: standard deviation.

**Table 3:** Autism symptoms, behavioral and psychopathological assessment.

Test/Participants	P1	P2	P3	P4	P6	P7	P8	P9	P10
**CARS 2**	22	NA	22.5	36.5 *	25.5	17	20.5	26	NA
**SRS**	Not clinical	NA	Non clinical	Social Awareness Social Cognition Social Communication Social Motivation Mannerisms SRS Total Score	Social Awareness Social Cognition Social Communication Social Motivation Mannerisms SRS Total Score	Social Awareness	Social Awareness Social Cognition Social Communication Social Motivation Mannerisms SRS Total Score	Social Awareness Social Cognition Social Communication Social Motivation Mannerisms SRS Total Score	NA
**CBCL**	Not clinical	NA	Not clinical	Attention Problem	Withdrawn/Depressed; Social Problems; Internalizing, Externalizing and Total Problems	Not clinical	Anxious/Depressed; Withdrawn/Depressed; Somatic Complaints; Social Problems; Thought Problems; Rule-Breaking Behavior; Aggressive Behavior; Internalizing, Externalizing and Total Problems	Anxious/Depressed; Withdrawn/Depressed; Social Problems; Thought Problems; Aggressive Behavior; Internalizing, Externalizing and Total Problems	Anxious/Depressed; withdrawn/Depressed; Somatic Complaints; Thought Problems; Internalizing, and Total Problems
**CPRS-R-L**	Not clinical	Cognitive problems/inattention; Anxious–shy; Social problems; Psychosomatic; DSM–IV inattentive; DSM–IV total	Not clinical	Not clinical	Cognitive problems/inattention; Anxious–shy; Perfectionism; Social problems; ADHD index; CGI restless–impulsive; CGI emotional lability; CGI total; DSM–IV inattentive; DSM–IV total	Perfectionism	Oppositional; Cognitive problems/inattention; Hyperactivity; Anxious–shy; Perfectionism; Social problems; Psychosomatic; ADHD index; CGI restless–impulsive; CGI emotional lability; CGI total; DSM–IV inattentive; DSM–IV hyperactive–impulsive; DSM–IV total	Oppositional; Cognitive Problems/inattention; Hyperactivity; Anxious–shy; Perfectionism; Social problems; Psychosomatic; ADHD index; CGI restless–impulsive; CGI emotional lability; CGI total; DSM–IV inattentive; DSM–IV hyperactive–impulsive; DSM–IV total	NA
**C-GAS**	Variable functioning (60)	NA	NA	NA	Moderate degree of interference in functioning (50)	Moderate degree of interference in functioning (50)	Moderate degree of interference in functioning in most social areas (47)	NA	Major impairment of functioning in several areas (40)
**Best Estimate Consensus Diagnosis**	Anxiety Disorder NOS (criteria not reached for Separation Anxiety Disorder and Social Phobia)	Anxiety Disorder NOS (criteria not reached for Generalized Anxiety Disorder)	Anxiety Disorder NOS (criteria not reached for Social Phobia)	Disruptive Behavior Disorder NOS	Disruptive Behavior Disorder NOS; Dysthymic Disorder	Generalized Anxiety Disorder (criteria not reached for Social Phobia)	Generalized Anxiety Disorder	Generalized Anxiety Disorder (criteria not reached for Social Phobia)	Generalized Anxiety Disorder (criteria not reached for Separation Anxiety Disorder and Social Phobia)
**Other Clinical Features**	Specific phobia (birds); stereotypes (hand flapping); Sporadic enuresis episodes; bruxism	Stuttering; Repetitive Movements	Selective Mutism Inhibition	Emotional dysregulation; hyperfagia; sleep talking; autistic features; alternate inhibition and social disinhibition (hugging non familiar people)	History of inhibition (actually social disinhibition); problem in motor regulation; repetitive behaviors; anxiety		Insistence on sameness; enuresis; emotional dysregulation; specific phobias; aggressive behaviors; social disinhibition (inappropriate sexual behavior; showing genitals); enuresis	Social disinhibition (hugging non-familiar people); emotional dysregulation; aggressive behaviors.	Behavioral oddities; Insistence on sameness; compulsive behaviors; imaginary friend

Only scores of SRS, CBCL, CPRS-R, ranging in borderline/clinical results are reported. C-GAS scores in parentheses ( ). Legend: P: Patient; *: above cut-off for autism; NA: not available; CARS2: Childhood Autism Rating Scale, Second Edition; SRS: Social Responsiveness Scale; CBCL: Child Behavior Checklist; CPR-S: Conners Rating Scales—Revised; C-GAS: Children’s Global Assessment Scale; NOS: not otherwise specified; NA: not available; ADHD: Attention Deficit Hyperactivity Disorder; CGI: Conners’ Global Index; DSM-IV: Diagnostic and statistical manual fourth edition.

**Table 4:** Presence/absence of anxiety/phobias, aggressive behaviors, separation anxiety/social inhibition, and repetitive behaviors/movements, disinhibition.

	P1	P2	P3	P4	P6	P7	P8	P9	P10	T
Anxiety/Specific Phobias	+	-	-	-	+	+	+	+	+	6/9
Separation Anxiety/Social Inhibition	+	+	+	+	+	+	+	+	+	9/9
Aggressive Behaviors	-	-	-	+	+	-	+	+	-	4/9
Repetitive Behaviors/Movements	+	+	-	-	-	-	-	+	+	4/9
Social Disinhibition	-	-	-	+	+	-	+	+	-	4/9

Legend: P: patient; +: presence of behavior; -: absence of behavior; T: Total.

## Supplementary Materials

The supplementary materials are available online at https://www.mdpi.com/2076-3425/10/11/839/s1.

## References

[1] Morris C.A., Thomas I.T., Greenberg F. (1993). Williams syndrome: Autosomal dominant inheritance. Am. J. Med. Genet..

[2] Sadler L.S., Robinson L.K., Verdaasdonk K.R., Gingell R. (1993). The Williams syndrome: Evidence for possible autosomal dominant inheritance. Am. J. Med. Genet..

[3] Pober B.R. (2010). Williams–Beuren Syndrome. N. Engl. J. Med..

[4] Collins R.T., Kaplan P., Somes G.W., Rome J.J. (2010). Cardiovascular Abnormalities, Interventions, and Long-term Outcomes in Infantile Williams Syndrome. J. Pediatr..

[5] Mason T.B., Arens R., Sharman J., Bintliff-Janisak B., Schultz B., Walters A.S., Cater J.R., Kaplan P., Pack A.I. (2011). Sleep in children with Williams Syndrome. Sleep Med..

[6] Meda S.A., Pryweller J.R., Thornton-Wells T.A. (2012). Regional brain differences in cortical thickness, surface area and subcortical volume in individuals with Williams syndrome. PLoS ONE.

[7] Nicita F., Garone G., Spalice A., Savasta S., Striano P., Pantaleoni C., Spartà M.V., Kluger G., Capovilla G., Pruna D. (2016). Epilepsy is a possible feature in Williams-Beuren syndrome patients harboring typical deletions of the 7q11.23 critical region. Am. J. Med. Genet. Part A.

[8] Berg J.S., Brunetti-Pierri N., Peters S.U., Kang S.-H.L., Fong C.-T., Salamone J., Freedenberg D., Hannig V.L., Prock L.A., Miller D.T. (2007). Speech delay and autism spectrum behaviors are frequently associated with duplication of the 7q11.23 Williams-Beuren syndrome region. Genet. Med..

[9] Mervis C.B., Klein-Tasman B.P., Huffman M.J., Velleman S.L., Pitts C.H., Henderson D.R., Woodruff-Borden J., Morris C.A., Osborne L.R. (2015). Children with 7q11.23 duplication syndrome: Psychological characteristics. Am. J. Med. Genet. Part A.

[10] Abbas E., Cox D.M., Smith T., Butler M.G. (2016). The 7q11.23 Microduplication Syndrome: A Clinical Report with Review of Literature. J. Pediatr. Genet..

[11] Merla G., Brunetti-Pierri N., Micale L., Fusco C. (2010). Copy number variants at Williams–Beuren syndrome 7q11.23 region. Hum. Genet..

[12] Morris C.A., Mervis C.B., Paciorkowski A.P., Abdulrahman O.A., Dugan S.L., Rope A.F., Bader P., Hendon L.G., Velleman S.L., Klein-Tasman B.P. (2015). 7q11.23 Duplication syndrome: Physical characteristics and natural history. Am. J. Med. Genet. Part A.

[13] Van Der Aa N., Rooms L., Vandeweyer G., Ende J.V.D., Reyniers E., Fichera M., Romano C., Chiaie B.D., Mortier G., Menten B. (2009). Fourteen new cases contribute to the characterization of the 7q11.23 microduplication syndrome. Eur. J. Med. Genet..

[14] Dixit A., McKee S., Mansour S., Mehta S., Tanteles G.A., Anastasiadou V., Patsalis P., Martin K., McCullough S., Suri M. (2012). 7q11.23 Microduplication: A recognizable phenotype. Clin. Genet..

[15] Prontera P., Serino D., Caldini B., Scarponi L., Merla G., Testa G., Muti M., Napolioni V., Mazzotta G., Piccirilli M. (2014). Brief Report: Functional MRI of a Patient with 7q11.23 Duplication Syndrome and Autism Spectrum Disorder. J. Autism Dev. Disord..

[16] Mari A., Amati F., Mingarelli R., Giannotti A., Sebastio G., Colloridi V., Novelli G., Dallapiccola B. (1995). Analysis of the elastin gene in 60 patients with clinical diagnosis of Williams syndrome. Hum. Genet..

[17] Guemann A.-S., Andrieux J., Petit F., Halimi E., Bouquillon S., Manouvrier-Hanu S., Van De Kamp J., Boileau C., Hanna N., Jondeau G. (2014). ELN gene triplication responsible for familial supravalvular aortic aneurysm. Cardiol. Young.

[18] Karmiloff-Smith A., Klima E., Bellugi U., Grant J., Baron-Cohen S. (1995). Is There a Social Module? Language, Face Processing, and Theory of Mind in Individuals with Williams Syndrome. J. Cogn. Neurosci..

[19] Karmiloff-Smith A., Brown J.H., Grice S., Paterson S. (2003). Dethroning the Myth: Cognitive Dissociations and Innate Modularity in Williams Syndrome. Dev. Neuropsychol..

[20] Vicari S., Caselli M.C., Gagliardi C., Tonucci F., Volterra V. (2002). Language acquisition in special populations: A comparison between Down and Williams syndromes. Neuropsychologia.

[21] Volterra V., Caselli M.C., Capirci O., Tonucci F., Vicari S. (2003). Early Linguistic Abilities of Italian Children With Williams Syndrome. Dev. Neuropsychol..

[22] Morris C.A. (2010). The behavioral phenotype of Williams syndrome: A recognizable pattern of neurodevelopment. Am. J. Med. Genet. Part. C Semin. Med. Genet..

[23] Alfieri P., Menghini D., Marotta L., De Peppo L., Ravà L., Salvaguardia F., Varuzza C., Vicari S. (2017). A comparison between linguistic skills and socio-communicative abilities in Williams syndrome. J. Intellect. Disabil. Res..

[24] Vivanti G., Hamner T., Lee N.R. (2018). Neurodevelopmental Disorders Affecting Sociability: Recent Research Advances and Future Directions in Autism Spectrum Disorder and Williams Syndrome. Curr. Neurol. Neurosci. Rep..

[25] Somerville M.J., Mervis C.B., Young E.J., Seo E.-J., Del Campo M., Bamforth S., Peregrine E., Loo W., Lilley M., Pérez-Jurado L.A. (2005). Severe Expressive-Language Delay Related to Duplication of the Williams–Beuren Locus. N. Engl. J. Med..

[26] Stinton C., Elison S., Howlin P. (2010). Mental Health Problems in Adults With Williams Syndrome. Am. J. Intellect. Dev. Disabil..

[27] Woodruff-Borden J., Kistler D.J., Henderson D.R., Crawford N.A., Mervis C.B. (2010). Longitudinal course of anxiety in children and adolescents with Williams syndrome. Am. J. Med. Genet. Part. C Semin. Med. Genet..

[28] Alfieri P., Scibelli F., Digilio M.C., Novello R.L., Caciolo C., Valeri G., Vicari S. (2020). Comparison of Adaptive Functioning in Children with Williams Beuren Syndrome and Autism Spectrum Disorder: A Cross-Syndrome Study.

[29] Velleman S.L., Mervis C.B. (2011). Children With 7q11.23 Duplication Syndrome: Speech, Language, Cognitive, and Behavioral Characteristics and Their Implications for Intervention. Perspect. Lang. Learn. Educ..

[30] Klein-Tasman B.P., Mervis C.B. (2018). Autism Spectrum Symptomatology Among Children with Duplication 7q11.23 Syndrome. J. Autism Dev. Disord..

[31] Mervis C.B., Morris C.A., Klein-Tasman B.P., Velleman S., Osborne L.R., Adam M.P., Ardinger H.H., Pagon R.A., Wallace S.E., Bean L.J.H., Stephens K., Amemiya A. (1993–2020). 7q11.23 Duplication Syndrome.

[32] Earhart B.A., Williams M.E., Zamora I., Randolph L.M., Votava-Smith J.K., Marcy S.N. (2016). Phenotype of 7q11.23 duplication: A family clinical series. Am. J. Med. Genet. Part A.

[33] Wechsler D. (2003). Wechsler Intelligence Scale for Children.

[34] Roid G.H., Miller L.J., Pomplun M., Koch C. (2013). Leiter International Performance Scale.

[35] Sparrow S.S., Cicchetti D., Balla D.A. (2005). Vineland Adaptive Behavior Scales.

[36] Harrison P.L., Oakland T. (2003). ABAS-II Adaptive Behavior Assessment System.

[37] Vicari S., Marotta L., Luci A. (2007). TFL Test Fono-Lessicale: Valutazione Delle Abilità Lessicali in età Prescolare.

[38] Kaplan E., Goodglass H., Weintraub S. (1983). Boston Naming Test.

[39] Marini A., Marotta L., Bulgheroni S., Fabbro F. (2015). Batteria per la Valutazione del Linguaggio in Bambini dai 4 ai 12 anni.

[40] Bello A., Caselli M.C., Pettinati P., Stefanini S. (2010). PinG: Parole in Gioco.

[41] Dunn L.M., Dunn L.M. (1997). Peabody Picture Vocabulary Test.

[42] Lancaster D.R.M. (2007). Prove di Valutazione della Comprensione Linguistica PVCL.

[43] Beery K.E., Buktenica N.A. (1997). The Beery–Buktenica Developmental Test of Visual–Motor Integration.

[44] Achenbach T.M., Rescorla L. (2001). Manual for the ASEBA School-Age Forms & Profiles.

[45] Conners C.K. (1997). Conners’ Rating Scales–Revised: User’s Manual.

[46] American Psychiatric Association (2000). Diagnostic and Statistical Manual of Mental Disorders.

[47] Kaufman J., Birmaher B., Brent D., Rao U., Flynn C., Moreci P., Williamson D., Ryan N. (1997). Schedule for Affective Disorders and Schizophrenia for School-Age Children-Present and Lifetime Version (K-SADS-PL): Initial Reliability and Validity Data. J. Am. Acad. Child. Adolesc. Psychiatry.

[48] Shaffer D., Gould M.S., Brasic J., Ambrosini P., Fisher P., Bird H., Aluwahlia S. (1983). A Children’s Global Assessment Scale (CGAS). Arch. Gen. Psychiatry.

[49] Constantino J.N., Davis S.A., Todd R.D., Schindler M.K., Gross M.M., Brophy S.L., Metzger L.M., Shoushtari C.S., Splinter R., Reich W. (2003). Validation of a Brief Quantitative Measure of Autistic Traits: Comparison of the Social Responsiveness Scale with the Autism Diagnostic Interview-Revised. J. Autism Dev. Disord..

[50] Schopler E., Van Bourgondien M.E., Wellman G.J., Love S.R. (2010). Childhood Autism Rating Scale.

[51] Castiglia L., Husain R.A., Marquardt I., Fink C., Liehr T., Serino D., Elia M., Coci E.G. (2018). 7q11.23 microduplication syndrome: Neurophysiological and neuroradiological insights into a rare chromosomal disorder. J. Intellect. Disabil. Res..

[52] Marler J.A., Sitcovsky J.L., Mervis C.B., Kistler D.J., Wightman F.L. (2010). Auditory function and hearing loss in children and adults with Williams syndrome: Cochlear impairment in individuals with otherwise normal hearing. Am. J. Med. Genet. Part C Semin. Med. Genet..

[53] Sammour Z.M., Gomes C.M., De Bessa J., Pinheiro M.S., Kim C.A., Hisano M., Bruschini H., Srougi M. (2014). Congenital genitourinary abnormalities in children with Williams–Beuren syndrome. J. Pediatr. Urol..

[54] Jackowski A.P., Rando K., De Araújo C.M., Del Cole C.G., Silva I., De Lacerda A.L.T. (2009). Brain abnormalities in Williams syndrome: A review of structural and functional magnetic resonance imaging findings. Eur. J. Paediatr. Neurol..

[55] Association on Intellectual and Developmental Disabilities (2010). Definition of Intellectual Disability. https://www.aaidd.org/intellectual-disability/definition#:~:text=Adaptive%20behavior%20is%20the%20collection,people%20in%20their%20everyday%20lives.

[56] Greer M.K., Brown F.R., Pai G.S., Choudry S.H., Klein A.J. (1997). Cognitive, adaptive, and behavioral characteristics of Williams syndrome. Am. J. Med. Genet..

[57] Mervis C.B., Klein-Tasman B.P., Mastin M.E. (2001). Adaptive Behavior of 4- Through 8-Year-Old Children With Williams Syndrome. Am. J. Ment. Retard..

[58] Mervis C.B., Klein-Tasman B.P. (2000). Williams syndrome: Cognition, personality, and adaptive behavior. Ment. Retard. Dev. Disabil. Res. Rev..

[59] Vicari S., Bates E., Caselli M.C., Pasqualetti P., Gagliardi C., Tonucci F., Evolterra V. (2004). Neuropsychological profile of Italians with Williams syndrome: An example of a dissociation between language and cognition?. J. Int. Neuropsychol. Soc..

[60] Heiz J., Barisnikov K. (2016). Visual-motor integration, visual perception and motor coordination in a population with Williams syndrome and in typically developing children. J. Intellect. Disabil. Res..

[61] Crespi B., Procyshyn T.L. (2017). Williams syndrome deletions and duplications: Genetic windows to understanding anxiety, sociality, autism, and schizophrenia. Neurosci. Biobehav. Rev..

[62] Dai L., Carter C.S., Ying J., Bellugi U., Pournajafi-Nazarloo H., Korenberg J.R. (2012). Oxytocin and Vasopressin Are Dysregulated in Williams Syndrome, a Genetic Disorder Affecting Social Behavior. PLoS ONE.

[63] Lee R., Ferris C., Van De Kar L., Coccaro E.F. (2009). Cerebrospinal fluid oxytocin, life history of aggression, and personality disorder. Psychoneuroendocrinology.

[64] Beitchman J.H., Zai C.C., Muir K., Berall L., Nowrouzi B., Choi E., Kennedy J.L. (2012). Childhood aggression, callous-unemotional traits and oxytocin genes. Eur. Child. Adolesc. Psychiatry.

[65] Malik A.I., Zai C.C., Abu Z., Nowrouzi-Kia B., Beitchman J.H. (2012). The role of oxytocin and oxytocin receptor gene variants in childhood-onset aggression. Genes Brain Behav..

[66] Thompson R.J., Parker K.J., Hallmayer J.F., Waugh C.E., Gotlib I.H. (2011). Oxytocin receptor gene polymorphism (rs2254298) interacts with familial risk for psychopathology to predict symptoms of depression and anxiety in adolescent girls. Psychoneuroendocrinology.

[67] Klein-Tasman B.P., Van Der Fluit F., Mervis C.B. (2018). Autism Spectrum Symptomatology in Children with Williams Syndrome Who Have Phrase Speech or Fluent Language. J. Autism Dev. Disord..

[68] Klein-Tasman B.P., Phillips K.D., Lord C.E., Mervis C.B., Gallo F.J. (2009). Overlap With the Autism Spectrum in Young Children With Williams Syndrome. J. Dev. Behav. Pediatr..

[69] Lord C., Rutter M., DiLavore P., Risi S., Gotham K., Bishop S. (2012). Autism Diagnostic Observation Schedule.

[70] Randall M., Egberts K.J., Samtani A., Scholten R.J., Hooft L., Livingstone N., Sterling-Levis K., Woolfenden S., Williams K. (2018). Diagnostic tests for autism spectrum disorder (ASD) in preschool children. Cochrane Database Syst. Rev..

