# Supplementary material for: 7q11.23 Microduplication Syndrome: Clinical and Neurobehavioral Profiling
Source: Brain Sci. 2020 Nov 11;10(11):839. doi: 10.3390/brainsci10110839 (PMC7697259; doi:10.3390/brainsci10110839)
Supplement: Supplementary file 1 [file brainsci-10-00839-s001.pdf]

Supplemental Material

1. **Characterization**

In the following paragraph, we provide further information on clinical features of children not reported in tables' paper. If not otherwise specified, scores from behavioral assessment are expressed in standard scores (Mean=100; SD= 15).

P1.

P1 is a second born of three siblings. Parents were non-consanguineous and healthy. Parents did not report any family history of previous diseases. Pregnancy was complicated by the risk of miscarriage (treated with progesterone from the end of the first trimester). Birth was at term by caesarian section. Birth weight was 3,595 g. At birth, he had difficulties in breast-feeding (poor suction); he required nasogastric tube feeding because of both swallowing and chewing difficulties (currently treated by a Language Speech Therapist specialized in dysphagia). He was fed with liquid food until the age of 3 years.

Sleep electroencephalogram (EEG), two-Dimensional Color-Doppler echocardiography and abdominal ultrasonography were normal.

At the time of genetic evaluation (7 years and 6 months), growth parameters were: weight 19 kg (below 3rd centile), height: 116.8 cm (3rd-10th centile). Physical examination displayed relative macrocephaly (OFC 53 cm, 75th centile), high forehead, elongated palpebral fissures, periorbital fullness, ears with additional crus of the antihelix and abnormally folded helix, microstomia with thin everted upper lip vermilion, webbed fingers and flat feet.

Developmental milestones were delayed. Independent walking was achieved at 17 months. The child has been exposed to bilingualism (Italian and Dutch). P1 has a normal sleep pattern. He has been toilet trained but he still has not acquired full bladder control. He shows teeth grinding and motor stereotypes.

The neurobehavioral profile was evaluated through standardized instruments at 7 years and 7 months. Expressive and receptive skills were tested by means of TFL (5). The scores were below average for chronological age (lexical production: raw score 33/45, <5°centile; lexical comprehension: raw score 9/45, <5 °centile). In spontaneous speech, the participant presented phonological difficulties.

Grammar comprehension was evaluated by means of PVCL (10). The participant obtained a score below the average for protocol 7 (age range: 7 years-7 years and 11 months).

P2

P2 is only child from non-consanguineous parents. A paternal aunt is affected by Down syndrome. The participants' mother and maternal grandfather presented psychomotor delay both on motor and language skills. The mother displayed facial features overlapping those in her son. Pregnancy was reported as regular. Perinatal history is unknown. The participant was admitted to the Genetic Department at 6 years because of psychomotor developmental delay and intellectual disability. Orchidopexy for bilateral cryptorchidism had been performed at 3 years of age. Evoked acoustic otoemissions (OAE) and EEG were regular. Ophthalmological evaluation revealed exophoria with normal fundus oculi. A cerebral ultrasound disclosed an enlargement of periencephalic cerebral spaces. Echocardiography disclosed mild ascending aortic dilatation. At 9 years he was diagnosed with hepatic steatosis with slight high levels of alanine aminotransferase (ALT) 45 > UI/L 5 – 40. Hepatic ultrasound revealed an increase of intracellular fat, suggesting severe liver steatosis. At 10 years, he started suffering from periodic migraine episodes.

At 6 years, auxological parameters were: weight 50 kg (>97th centile), height 152 cm (90th-97th centile), OFC 54.3 cm (50°-75° centile). Physical examination disclosed brachycephaly, deep set eyes, telecanthus, medial flaring of the eyebrows, horizontal crus of helix, high nasal bridge, short philtrum, thin upper lip. Hands with tapering fingers, shawl scrotum, one cafè-au-lait spot on the left limb, pes planus.

The neurobehavioral profile was evaluated through standardized instruments at 9 years and 10 months. The cognitive profile was assessed using WISC-IV (1). The Full Scale Intelligence Quotient (FSIQ) was below the average range (FSIQ = 47); the four indexes were distributed as follow: Verbal Comprehension Index (VCI) = 66, Perceptual Reasoning Index (PRI) = 56, Working Memory Index (WMI) = 64, Processing Speed Index (PSI) = 53.

The receptive vocabulary was measured by using the PPTV (9), while expressive language by Boston Naminig Test- BNT (6). The child obtained a score below the mean in both PPTV (LQ: 78) and BNT (the score was two SD below the mean for his school age; raw scores 26/40).

Grammar comprehension was evaluated by means of PVCL (9). The participant obtained a score on average for the 6/7 protocol (age range 7 years-7 years and 11 months).

P3

P3 is the second born girl of non-consanguineous with a negative family history. Pregnancy was regular and she was born at term. Amniocentesis was performed due to a worrisome prenatal screening test and revealed a normal karyotype. Birth weight was 2.615 gr. Apgar scores were 9-9 at 1st and 5th minute, respectively. P2 presented neonatal bradycardia and muscular hypotonia. Periventricular echogenicity on neonatal cranial ultrasound was evident. OAE, Brainstem Auditory Evoked Potential (BAEP) and Color-Doppler echocardiography were normal. EEG revealed modest abnormalities in the anterior central spikes. A Brain MRI performed at 3 years disclosed atrophy of the entorhinal cortex with enlargement of the third and lateral ventricles, thin corpus callosum, regions of porencephaly in the periventricular white matter.

Physical examination disclosed a generalized hypotonia more pronounced in the lower limbs, joint hyperlaxity, horizontal eyebrows, synophrys, high nasal bridge with bulbous nose, asymmetric low set and posteriorly rotated ears (the right ear being more pronounced), thin upper lip vermilion, short neck, pectus excavatum, mild hypertrichosis on the trunk, flat feet. At 7 years, growth parameters were within the low normal ranges with weight 17 kg (10th centile), height 108 cm (10th centile), OFC 49 cm (25th centile).

Psychomotor development was delayed. She walked at 30 months and could climb up and down the stairs only if supported. She was able to pronounce only single words, she has a contextual comprehension for simple orders and showed pointing. Sleep pattern was normal.

The neurobehavioral profile was evaluated through standardized instruments at 5 years and 7 months. Receptive and expressive skills were assessed by means of PinG (8). Receptive vocabulary was on average for the 37 months, whilst expressive skills we on average for 23 months.

Grammar comprehension was evaluated by means of PVCL (10). The participant did not reach minimum criteria for the protocol 3 (age range: 3 years – 3 years and 11 months).

P4

P4 was born at term after uncomplicated pregnancy. Birth weight was 3.470 gr. OAE and audiometric testing were negative. Fundus oculi and renal ultrasound were normal. Growth was within normal range for the first year of life, with subsequent increased of weight due to high caloric diet. The patient is in follow-up because of asthmatic bronchitis. Cardiological evaluation with ECG was negative. He underwent orchidopexy for bilateral cryptorchidism (right at 2 and left at 6 years).

Physical examination indicated macrocephaly, palpebral ptosis (more pronounced in the left eye), elongated and down slanted palpebral fissures, prognathism, long chin with horizontal crease, low-set posteriorly rotated ears with thickened earlobes (Figure 1). At the age of 6 years and 3 months weight was 37.9 kg (10th centile), height was 126.5cm (97th centile), OFC 55,5cm (>97th centile).

The boy has a psychomotor delay. He walked independently at 16 months. He is currently able to pronounce two words' simple sentences. He has an emotional dysregulation, improving over the time. He is currently living in a foster home with his mother. His father died at 32 years because of testicular seminoma.

The neurobehavioral profile was evaluated through standardized instruments at 5 years and 6 months. Receptive vocabulary was evaluated by means of TFL (5). The score was on average for 42 month (raw scores 29/45, <5°centile). Expressive vocabulary was measured by means of PinG (8). The score was on average for 30 months.

Grammar comprehension was evaluated by means of PVCL (10). Participant was on average for the protocol 3 (age range: 3 years – 3 years and 11 months).

P5

P5 is the second child of non-consanguineous healthy parents with a negative family history. The father displays mild dysmorphic features including low posterior hair line and downslanting palpebral fissures. He has not performed an echocardiogram. Maternal echocardiogram was normal. An atrial septal defect was surgically repaired in a paternal aunt and her daughter.

Amniocentesis, performed in the participant's pregnancy complicated by pulmonary valve dysplasia, disclosed a normal karyotype. P5 was born at 38 weeks of gestational age. Birth weight was 3.800 gr, birth length 48 cm, OFC 42 cm. Apgar scores were 8-9. At birth, a cerebral ultrasound revealed a megacisterna magna and periventricular white matter hyperintensities, which were not visible at a following examination. Color-Doppler echocardiography disclosed a patent foramen ovale and pulmonary valve dysplasia. Audiometric testing was normal.

Physical evaluation included downslanting palpebral fissures, microstomia, micrognathia, everted upper lip vermilion, thickened helices, intrarotated gait, tibia vara, lumbar hyperlordosis.

She walked independently at 14 months. Language was delayed. Neurological examination was normal. She has an appropriate social motivation and no dysfunctional behavior. Language was delayed (at 3 years she pronounced around 50 words). She was able to compose two simple words sentences. The neurobehavioral profile was not evaluated through standardized instruments.

P6

P6 was born from a pregnancy complicated by reactivation of the mother's Crohn disease (surgically treated during and after the pregnancy). P6 was born at 38 week of gestational age. Fetal distress was documented by fetal heart rate deceleration. Birth weight was 2.980 g. Cardiac evaluation was normal. He had an irregular sleep pattern until age of 4 years with agitated insomnia. Pes planus was treated with orthotic arch supports. He underwent adenoidectomy at 8 years and presented a mild conductive hearing loss due to tubal stenosis. He suffered from bronchospasm treated with corticosteroids. At time of genetic evaluation at 9 years and 10 months growth was within normal ranges and at 9 years and 10 months weight was 37 kg (59th-75th centile), height 131.5 cm (10th-25th centile).

Psychomotor milestones were delayed. He walked at 24 months. He displayed an aggressive behavior.

The neurobehavioral profile was evaluated through standardized instruments at 12 years and 3 months. The cognitive profile was assessed using WISC-IV (1). The Full Scale Intelligence Quotient (FSIQ) was in the borderline range (FSIQ = 75); the four indexes were: VCI = 88, PRI= 76, WMI = 91, PSI = 71.

Receptive and expressive skills were measured by means of BVL (7); comprehension (raw scores 41/42) and production (raw score 66/67) were on average for the last age range available in the tool (11 years and 6 months – 11 years and 11 months).

In the receptive grammar subtest (BVL) (7), the participant obtained a score two SD below the mean (raw scores 33/40).

The visual-motor integration ability score (11) was below the mean (Standard Score 58); in motor coordination subtest, the participant's score was below the mean (Standard Score 63), while in the visual perception subtest score was only one SD below the mean (Standard Score 84).

P7

P7 is a second child of healthy non-consanguineous parents. P7 has an older brother (14 years) diagnosed with ADHD and learning disability. A paternal aunt was diagnosed with bipolar disorder. She was born at term after an uncomplicated pregnancy by spontaneous delivery. Birth weight was 3.650 g. The perinatal period was regular. OAE and audiometric testing were normal. At age of 8 years, echocardiography revealed patent ductus arteriosus 1.5 mm in diameter with mild left-to-right shunt and mild tricuspid insufficiency. Abdominal ultrasound was negative. At physical evaluation (7 years and 11 months) weight was 28.1 kg (50th-75th centile), height 125.5 (25-50th centile), and OFC 52 cm (50th centile). Clinical examination revealed broad forehead, horizontal eyebrows, telecanthus, elongated palpebral fissures, bulbous nose, short philtrum, short neck, widely spaced teeth, hyperlaxity (Figure 1).

P7 had a motor delay. She was able to sit unsupported at 11 months and walked independently at 19 months. She babbled at 4 years. She pronounced her first words after age of 5 years. She has a normal sleep pattern. Brain MRI and EEG at 4 years and 5 months were normal.

The neurobehavioral profile was evaluated through standardized instruments at 9 years and 2 months. Receptive and expressive skills were measured by means of BVL (7). The receptive vocabulary subtest score was one SD below the mean (raw score 56/67), the expressive vocabulary score two SD below the mean (raw score 15/42) as the morph-syntactic production subtest score (raw score 10/20).

In the receptive grammar subtest (BVL) (7), the participant obtained a score two SD below the mean for her chronological age (raw score 30/40).

P8

P8 is the only child of non-consanguineous parents with a negative family history. During pregnancy, the mother took progesterone for 2 months without complications. Birth was at 38 week of gestational age by caesarean section. Birth weight was 3.590 kg, length 52 cm, OFC 35 cm. Apgar score were 9-10. The perinatal period was unremarkable. OEA, BAEP, EEG, Color-Doppler echocardiography and renal ultrasound were negative. Ophthalmological evaluation disclosed hypermetropia, and astigmatism with a normal fundus oculi. Auxological parameters were within normal range. At 2 years, weight was 13.70 kg (75th – 97th centile), height 88 cm (25th – 50th centile), OFC 51cm (75th centile). Physical examination revealed brachycephaly, deep set eyes, short palpebral fissures, prominent nasal septum with bulbous nasal tip and overhanging columella, short philtrum. He walked independently at 21 months. He presented episodic aggressive behaviors and has a regular sleep pattern.

The neurobehavioral profile was evaluated through standardized instruments at 7 years and 5 months.

The cognitive profile was assessed using WISC-IV (1). FSIQ was below the average (FSIQ = 65); the four indexes were distributed as follow: VCI = 86, PRI= 80, WMI = 61, PSI = 59.

Receptive skills were measured by means of PPTV (9). The score was on average for the range 4 years and 3 months – 4 years and 8 months. Expressive skills were assessed through BNT (Kaplan et al., 1983). The score was on average for his school age.

Grammar comprehension was evaluated by means of PVCL (10). The participant obtained a score below the mean for protocol 4B (age range: 4 years and 6 months – 4 years and 11 months).

The visual-motor integration ability score (11) was in average for the chronological age (89), but the visual perception and motor coordination subtests scores were two SD below the mean (respectively 68 and 60).

P9

P9 is first of three siblings. The father is affected by the same dup7q11.23. The P9's father is 187 cm tall, with macrocephaly (OFC 61 cm, >97th centile), and manifests learning disabilities. He attended school with support.

P9 was born after a pregnancy complicated by abnormal results of prenatal screening test. He was born at 41 weeks of gestational age by spontaneous vaginal delivery. Birth weight 3.220 g, length 50 cm, OFC 34 cm. The perinatal period was regular. OEA was normal. At birth, echocardiography revealed a small patent ductus arteriosus that resolved, spontaneously. Feeding and growth were normal. At 6 months, he performed brain MR and EEG because of the suspect of flexion spasms, which were subsequently excluded. A cytomegalovirus infection was documented by presence of the virus in urine.

At 6 years and 8 months weight was 24.8 kg (75th percentile), height 127 cm (97th percentile), OFC 52.5cm (75th percentile). Physical evaluation revealed synophrys, hypertelorism, divergent strabismus, elongated palpebral fissures, large ears, thick nostrils, thin lips (Fig. 1). Developmental milestones were delayed; he walked alone at 18 months.

The neurobehavioral profile was evaluated through standardized instruments at 7 years and 8 months.

Receptive and expressive skills were assessed by means of TFL (5). Score was on the average for the age of 39 months (raw score 30/45, <5 °centile). Expressive level was not computable because of the child's non compliance.

In the PVCL receptive grammar test he was below the mean for protocol 4B (age range: 4 years and 6 months – 4 years and 11).

P10

P10 is the only child of non-consanguineous parents, born from caesarian section at 38 weeks of gestational age due to maternal intervertebral disc disease. The mother presented stutter and strabismus. Pregnancy was complicated by gestosis. Birth weight was 2.540 g. Apgar scores were 7-10 at 1st and 5th minutes. P10 was hospitalized in the neonatal intensive care because of sepsis, hypotonia with suction difficulties and bradypnea. He presented a renal pelvis dilatation (spontaneously solved). Echocardiography showed left ventricular hypertrabeculation, mild aortic insufficiency. At 11 years and 10 months, blood tests for thyroid function (TSH, FT4) were normal. A thyroid ultrasound performed at 11 years disclosed normal morphology of the thyroid gland with a homogeneous appearance and a reduction of size of both lobes. Bone age assessed with the Greulich-Pyle methods was advanced (1 year and 8 months).

At 11 years and 10 months, weight was 62 Kg (97th centile), height 151.2 cm (50-75th centile). Physical examination displayed severe obesity with truncal distribution. He presented elongated palpebral fissures,

large incisors, short neck, low anterior and posterior hairline. Psychomotor development was delayed; he walked independently at 3 years. At 4 years and 10 months, brain MRI revealed a thinning of the corpus callosum and an arachnoid cyst in the left median and paramedian cerebellar portion. EEG was negative.

The neurobehavioral profile was evaluated through standardized instruments at 11 years and 3 months. Receptive skills were measured by PPTV (9). Score was on average for the range 4 years and 3 months – 4 years and 8 months age range. Expressive skills were evaluated by BNT (6). The participant was on average for first grade of elementary school.

Grammar comprehension was evaluated by means of PVCL (10). The participant did not reach criteria for protocol 4B (4 years and six months – 4 years and 11 months age range).

References

1. Wechsler, D.; *Wechsler Intelligence Scale for Children-fourth edition (WISC-IV);* Psychological Corporation: San Antonio, TX, USA, **2003.**

2. Roid, G. H.; Miller, L. J.; Pomplun, M.; Koch, C.; *Leiter International Performance Scale, Third Edition (Leiter-3)*; Western Psychological Services: Los Angeles, CA, USA, **2013.**

3. Sparrow, S.S.; Cicchetti, D.; Balla, D.A. *Vineland Adaptive Behavior Scales-2nd edition manual*; NCS Pearson Inc: Minneapolis, MN, USA, **2005.**

4. Harrison, P.L.; Oakland, T. *ABAS-II Adaptive Behavior Assessment System, 2nd ed*.; Harcourt Assessment, Inc, San Antonio, TX, USA, **2003.**

5. Vicari, S.; Marotta, L.; Luci. A. *TFL Test Fono-lessicale.: Valutazione delle abilità lessicali in età prescolare*. Edizioni Erickson: Trento, Italy, 2007.

6. Goodglass, H.; Kaplan, E.; Weintraub, S. *Boston naming test*. Lea & Febiger: Philadelphia, PA, USA,**1983.**

7. Marini, A.; Marotta, L.; Bulgheroni, S.; Fabbro, F. *Batteria per la Valutazione del Linguaggio in Bambini dai 4 ai 12 anni*. Giunti OS: Firenze, Italy, **2015**.

8. Bello, A.; Caselli, M.C.; Pettinati, P.; Stefanini, S. *PinG: Parole in Gioco*. Giunti O.S.: Florence, Italy, **2010**

9. Dunn, L.M.; Dunn, L.M. *Peabody picture vocabulary test— Third edition*. American Guidance Services, Inc: Circle Pines, MN, **1997**.

10. Rustioni Metz Lancaster, D. *Prove di Valutazione della Comprensione Linguistica PVCL*. Giunti Psychometrics: Firenze, Italy, **2007**.

11. Beery, K.E.; Buktenica, N.A. *The Beery–Buktenica developmental test of visual–motor integration*. NCS Pearson: Minneapolis, MN, **1997**.

12. Achenbach, T. M.; Rescorla, L. *Manual for the ASEBA School-Age Forms & Profiles*. University of Vermont: Burlington, VT, **2001**.

13. Conners, C.K. *Conners' rating scales–revised: user's manual*. MultiHealth Systems, Incorporated: North Tonawanda, NY, **1997**.

14. American Psychiatric Association. *Diagnostic and statistical manual of mental disorders, 4th edn., text rev.* American Psychiatric Association: Washington, DC, **2000**.

15. Kaufman, J.; Birmaher, B.; Brent, D.; Rao, U.; Flynn, C.; Moreci, P.; Williamson, D.; Ryan, N. Schedule for Affective Disorders and Schizophrenia for School-Age Children-Present and Lifetime Version (K-SADS-PL): initial reliability and validity data. *J. Am. Acad. Child. Adolesc. Psychiatry*. **1997**;36(7):980-988. doi:10.1097/00004583-199707000-00021

16. Shaffer, D.; Gould, M.S.; Brasic, J.; Ambrosini, P.; Fisher, P.; Bird, H.; Aluwahlia, S. A children's global assessment scale (CGAS). Arch. Gen. Psychiatry. **1983**;40(11):1228-1231. doi:10.1001/archpsyc.1983.01790100074010
